# Epigenetic Regulation of Angiogenesis in Development and Tumors Progression: Potential Implications for Cancer Treatment

**DOI:** 10.3389/fcell.2021.689962

**Published:** 2021-09-06

**Authors:** Veronica Mãdãlina Aspriţoiu, Ileana Stoica, Coralia Bleotu, Carmen Cristina Diaconu

**Affiliations:** ^1^Faculty of Biology, University of Bucharest, Bucharest, Romania; ^2^Romanian Academy, Stefan S. Nicolau Institute of Virology, Bucharest, Romania

**Keywords:** epigenetic regulation, angiogenesis, development, tumors progression, cancer treatment

## Abstract

Angiogenesis is a multi-stage process of new blood vessel development from pre-existing vessels toward an angiogenic stimulus. The process is essential for tissue maintenance and homeostasis during embryonic development and adult life as well as tumor growth. Under normal conditions, angiogenesis is involved in physiological processes, such as wound healing, cyclic regeneration of the endometrium, placental development and repairing certain cardiac damage, in pathological conditions, it is frequently associated with cancer development and metastasis. The control mechanisms of angiogenesis in carcinogenesis are tightly regulated at the genetic and epigenetic level. While genetic alterations are the critical part of gene silencing in cancer cells, epigenetic dysregulation can lead to repression of tumor suppressor genes or oncogene activation, becoming an important event in early development and the late stages of tumor development, as well. The global alteration of the epigenetic spectrum, which includes DNA methylation, histone modification, chromatin remodeling, microRNAs, and other chromatin components, is considered one of the hallmarks of cancer, and the efforts are concentrated on the discovery of molecular epigenetic markers that identify cancerous precursor lesions or early stage cancer. This review aims to highlight recent findings on the genetic and epigenetic changes that can occur in physiological and pathological angiogenesis and analyze current knowledge on how deregulation of epigenetic modifiers contributes to tumorigenesis and tumor maintenance. Also, we will evaluate the clinical relevance of epigenetic markers of angiogenesis and the potential use of “epi-drugs” in modulating the responsiveness of cancer cells to anticancer therapy through chemotherapy, radiotherapy, immunotherapy and hormone therapy as anti-angiogenic strategies in cancer.

## Introduction

Angiogenesis is a multi-stage process defined as new blood vessels development that originates from pre-existing vessels, which usually grow toward an angiogenic stimulus (Adair and Montani, 2010). The process is essential for tissue maintenance and homeostasis during embryonic development and adult life as well as tumor growth. Under physiological conditions, angiogenesis is involved in physiological processes, such as placental development, cyclic regeneration of endometrium, wound healing and repairing the damage inflicted by ischemia or cardiac failure (Ferrara and Alitalo, 1999). However, aberrations of the phenomenon may constitute the pathogenetic basis of diseases such as cancer, an essential condition for the early stages of tumoral maintenance and development of vascular networks that support metastases in the advanced stages of the disease. Angiogenesis is necessary for cancer growth and metastasis and, therefore, an imperative goal for cancer research. In cancer biology, vessels network are crucial for nutrients and oxygen supply for the tumor, and new blood circuits are essential for the maintenance of tumor cells, ensuring nutrients and removing metabolic waste from tumor sites (Tonini et al., 2003). The regulatory mechanisms in physiological angiogenesis are coordinated, well balanced, and strictly regulated by pro and anti-angiogenic factors. In contrast, tumor angiogenesis is characterized by the excess of pro-angiogenic factors that lead to uncoordinated endothelial cell (EC) proliferation and supportive cell migration (Pozzi and Zent, 2009).

Unlike normal blood vessels, the structure and function of the tumor vasculature are abnormally characterized by the affectation of pericytes and basement membrane (BM), small vessel diameter, heterogeneous vascular density and high permeability to large molecules (Tonini et al., 2003). These abnormalities contribute to an abnormal microenvironment, characterized by the pressure generated in growing tumors which compresses the intratumoral blood and lymphatic vessels leading to an inadequate blood supply, interstitial hypertension, hypoxia, and acidosis (Padera et al., 2004). On the other hand, the hypoxic microenvironment is a precondition for tumor drug resistance. A possible explanation of resistance to radiotherapy may be that oxygen is mandatory for the cytotoxic effects of ionizing radiation. Furthermore, resistance to chemotherapy is often characterized by an insufficient drug supply in the treated tumor (Vaupel et al., 2001). Specific anti-angiogenic agents can adjust tumor angiogenesis, normalize of tumor vasculature, and improve blood flow and tumor perfusion, reducing vascular permeability and interstitial fluid pressure (Jain, 2001). This hypothesis was supported by several preclinical and clinical studies in which anti-angiogenic therapy was combined with chemotherapy and/or radiotherapy for glioblastoma, ovarian, breast, colorectal, and pancreatic cancer. Unfortunately for these patients, the progression-free survival and overall survival were only for few months, and new therapeutic strategies are under exploration to improve survival for patients with cancer (Montemagno and Pagès, 2020).

Considering that all organisms need to adapt quickly to environmental changes, epigenetic regulation of genes is the mechanism through which organisms rapidly adapt to these changes. Thus, the analysis of the epigenetic status of different modified genes, chromatin regions, histone and miRNA in various pathologies was developed, leading to the discovery of many useful epigenetic biomarkers. For example, the DNA methylation signature in a tumor sample may have additional medical value by being used as prognostic and diagnostic biomarkers in different cancer types. Using methylation data, it was concluded that patients with a higher methylation grade at CpG islands who showed aberrant hypermethylation at target genes had poorer results than patients with a lower methylation grade. Hence, methylation can be a good indicator of survival prediction and of response to therapies (Shen et al., 2010). In addition, it has been observed that epigenetic agents inhibit the development of latent cancer cells that express anti-angiogenic genes. These genes are reduced during switching to active growth by changes in DNA methylation and histone changes (Lyu et al., 2013).

In this review, we have summarized information about the non-pathological angiogenic process but also the implications of angiogenic agents in the development and progression of tumors, by addressing vascular cooption and intussusception, sprouting angiogenesis and/or vasculogenic mimicry to meet the needs of supplementary vasculature. Moreover, we have presented important epigenetic agents and their role in carcinogenesis that can be used as treatment targets in various pathologies. Epigenetic changes have reversible effects and in general epigenetic agents have lower toxicity, having potential in antiangiogenic treatments or as adjuvants.

## Angiogenesis Mechanisms

**FIGURE 1:**
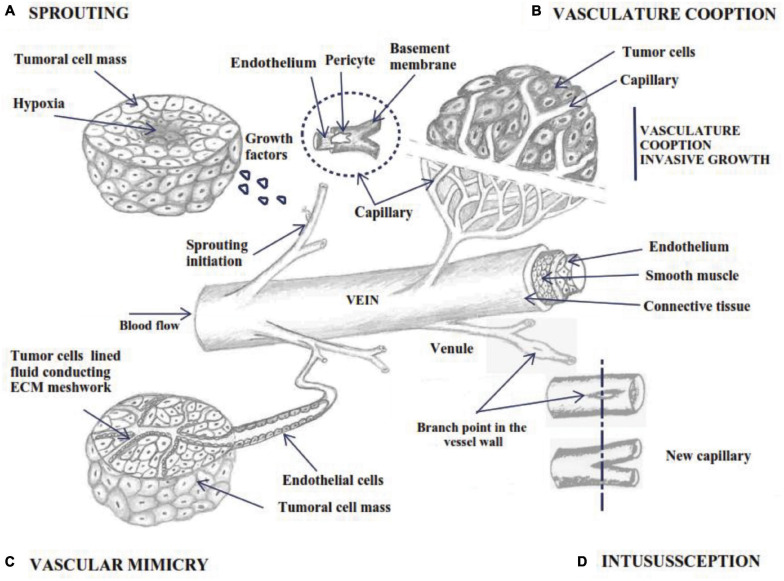
Schematic outline of angiogenesis by 4 different mechanisms; **(A)** sprouting angiogenesis: bud initiation from capillary wall under growth factor signaling; **(B)** vascular cooption of nearby tissue vessels as its own source of nutrients; **(C)** vascular mimicry – the tumor cells adopt endothelial phenotype and form a vasculogenic-like network **(D)** vessel intussusception involves the splitting of pre-existing vessels into two new vascular structures.

**FIGURE 2:**
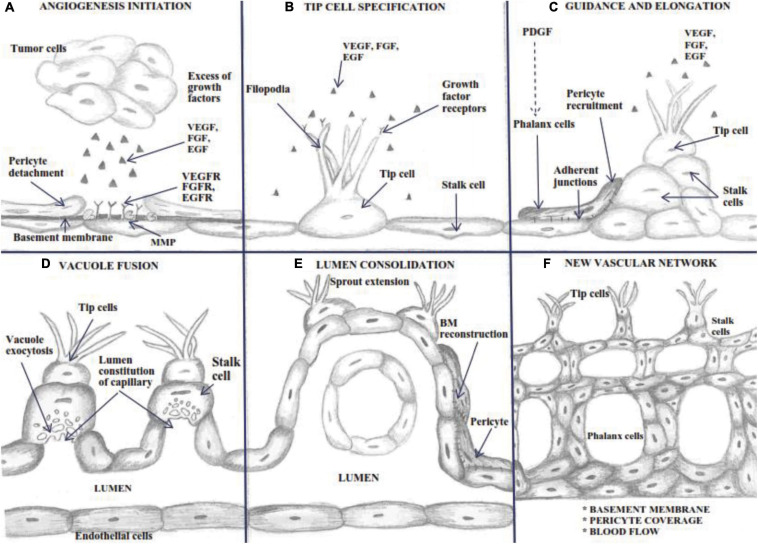
Schematic outline of the Sprouting process. Angiogenesis is a complex process involving: **(A)** a basement membrane alteration produced by MMP, which expose growth factors receptors, characterized by a dilated mother vessel projecting ECs into the connective tissue; **(B)** tip cell specification and filopodia extension determined by growth factors amount-dependent arising from the growing capillary bud; **(C)** ECs migrate in parallel establishing a lumen and sealed by integral endothelial junctions and pericyte recruitment; **(D)** stalk cells display vacuole exocytosis for lumen constitution; **(E)** polarized ECs continuously deposit the basement membrane, and the proliferating pericytes migrate through the basement membrane of the capillary sprout, resulting in the entire constitution of the new vessel wall; **(F)** complete BM reconstitution and the restoration of blood flow.

Angiogenesis is described as microvascular growth under an angiogenic stimulus (Figure 1A; Risau and Flamme, 1995). As a general mechanism, the process involves a structural alteration of the basement membrane (BM) and dilated vessel. After partial degradation of the basement membrane, endothelial cell migrates maintaining their basal-luminal polarity forming interendothelial junctions. The new growing vessel elongates toward angiogenic factors while BM is deposited continuously by the endothelial cell, and pericytes are recruited for the new vessel coverage (Figure 2A; Paku and Paweletz, 1991).

Several ways to develop new blood vessels have been described: sprouting angiogenesis, intussusception, vascular mimicry and cooption and using endothelial progenitor cells or angioblasts. Sprouting angiogenesis is the process by which specialized endothelial tip cells migrate from preexisting vessels and invade surrounding avascular tissue. The process starts with sprouts composed of specific and proliferative endothelial cells (ECs), which usually grow toward an angiogenic stimulus (Figure 1A; Adair and Montani, 2010; Groppa et al., 2018).

Non-sprouting vessels can be intussusceptive or splitting angiogenesis (Figure 1D), through which the intraluminal walls are divided longitudinally from the pre-existing vessels into new smaller ones (Gianni-Barrera et al., 2011; Ribatti and Crivellato, 2012). In addition, two models were observed during tumor development: vascular mimicry and vascular cooption with implication on tumor progression (Figures 1B,C). It was observed that angioblasts isolated from human peripheral blood and differentiated in ECs *in vitro*, have the ability to be incorporated into active sites of angiogenesis. This may be an important fact in the normalization of tumor vasculature, supplementing vessel growth in therapeutic angiogenesis (Asahara et al., 1997).

### Quiescence and Sprouting: Regulation of Tip and Stalk Cell

Endothelial cells cover the vessels’ inner surface, an intricate network nourishing all organism tissues. In embryonic development, *de novo* vessels appear by assembling endothelial precursors that differentiate to form a primitive vascular system (vasculogenesis) (Potente et al., 2011). Upcoming vessel sprouting by angiogenesis creates new blood vessels that originate from pre-existing ones and requires pericytes and vascular smooth muscle cells for covering nascent EC (Adair and Montani, 2010; Carmeliet and Jain, 2011). In healthy adults, vessels are quiescent, but their constitutive ECs maintain their plasticity to sense and respond to angiogenic stimuli. Thus, under pro-angiogenic signals influences, ECs become motile and invade the surrounding sites (Carmeliet and Jain, 2011). Tip cells, the ECs located at the tip of the sprout, are migratory and polarized cells with minimal proliferation rate, leading to new-formed vessels. Using their many phyllopods, they sense endogenous stimuli from the environment and guide the angiogenic sprout toward the direction of stimuli (Figures 2B,C; del Toro et al., 2010).

In contrast, stalk cell proliferates during sprout extension behind the leading tip cell and forms the nascent vascular lumen cells by maintaining their position and connection to the parent vasculature. Stalk cells shape the branches and organize the vascular lumen of the new routes near the sprout (Gerhardt et al., 2003), constitute cell-cell junctions with adjacent cells, and synthesize components for the basement membrane (Phng and Gerhardt, 2009). During maturation, stalk cells transform into phalanx cells (De Spiegelaere et al., 2012). The proliferation rate of the phalanx cells is slower than the stalk cells. They resemble resting ECs, but forms the basement membrane continuously and strengthens the tight junctions forming a tight barrier between the blood and the surrounding tissue (De Smet et al., 2009).

Quiescent ECs are non-proliferating cells with long half-lives, which are maintained by factors like vascular endothelial growth factor (VEGF), NOTCH, angiopoietin-1 (ANG-1) and fibroblast growth factors (FGFs) (Carmeliet and Jain, 2011). The quiescent phenotype is adopted for vessel integrity through increased cell adhesion. Phalanx cells are immobile cells, which line the newly established perfused vessel. They are closely connected by tight junctions and adherens junctions, strengthening the blood vessel wall and forming a lumenized barrier between blood and surrounding tissues that control fluid exchange and immune cell infiltration (Potente et al., 2011).

Cell-cell adhesion between ECs and neighboring cells is regulated at the adherent junctions at the endothelium level by transmembrane adhesive proteins, VE-cadherin N-cadherin, as well as claudin, occludin, nectins, and junctional adhesion molecules (JAMs). Tight junction molecules maintain and regulate paracellular permeability (Gavard and Gutkind, 2008). VE-cadherin interacts with the cytoskeleton and controls EC adhesion by solidifying the wall or facilitating EC separation and movement. In a complex with VEGFR2, VE-cadherin sustains EC quiescence by recruiting phosphatases, as VE-PTP (Vascular Endothelial Protein Tyrosine Phosphatase) and DEP-1, that remove the phosphate group from the VEGFR2 level, thus limiting VEGF signaling. In addition, activation of TIE2 by ANG1 protects the vessels wall from VEGF-induced cellular mobility by blocking the capability of VEGF to induce VE-cadherin endocytosis (Potente et al., 2011). The main function of VEGF functions in angiogenesis are presented in Box 1.

**Box 1. The functions of the VEGF family members.**

Each of the six homologous genes from the vascular endothelial growth factors family, the VEGF-A, VEGF-B, VEGF-C, VEGF-D and placental growth factors (PlGF1–4), resulting from alternative splicing, plays a significant role in various contexts of vascular growth, from embryonic vasculogenesis to blood and lymphatic angiogenesis in parenchymal tissues (Ferrara, 2004). VEGF-A is the main component of the family. It plays a fundamental role in physiological and pathological angiogenesis by signaling through the VEGF-2 receptor (VEGFR-2, also known as FLK1). VEGFR-2 is a tyrosine kinase receptor expressed by ECs, positively conducting mitogenic and chemotactic signaling in this type of cells correlated with VEGF concentrations (Ribatti and Crivellato, 2012). VEGFR-1 is predominantly expressed in stalk cells, with major importance in guiding and inhibiting tip cell formation, using NOCH signaling that reduces the availability of VEGF ligand and prevents external migration of the tip cell (Chappell et al., 2009). VEGFR-3 is essential in vasculogenesis and is strongly expressed in the leading tip cell, being a fundamental regulator of the development of new lymphatic and blood vessels. VEGFR-3 is tip cell marker. In stalk cell it is down-regulated by NOTCH signaling pathway during sprouting angiogenesis (Ribatti and Crivellato, 2012). Also, VEGFR3 is highly expressed in endothelial lymphocytes. In addition to its promotion of vascular ECs proliferation, VEGF has also been reported to promote vascular permeability through the phosphorylation of VE-cadherin conditioned by VEGF-induced initiation of Rac, which weaken cell-cell interaction in ECs (Gerwins et al., 2000).

Initiation of sprouting involves endothelial cell specification into three different subtypes bearing different morphologies and functional properties: filopodia-rich migratory tip cells, highly proliferating stalk cells and quiescent phalanx cells (Table 1). Sprouting angiogenesis is initiated through oxygen-sensing mechanisms that detect a hypoxia level and initiates the formation of new blood vessels that meet the metabolic requirements of tumor or somatic cells (Figure 1A). Oxygen levels are heterogeneous inside of solid tumors and can range from 2–0.1% O_2_ (Hammond et al., 2014). These cells respond to the hypoxic environment by producing and secreting pro-angiogenic growth or proteolytically liberated angiogenic factors from the extracellular matrix (ECM) molecules (Adair and Montani, 2010).

**TABLE 1:** The main cells involved in angiogenesis process.

Cells types	Main function during angiogenesis
Endothelial cells	Line blood vessels having a quiescent behavior in normal conditions
Tip cells	Cells leading to the formation of new vessel branches. They are motile, invasive, highly polarized with a large number of long filopodial protrusions which can extend, lead and guide endothelial sprouts in their environment
Stalk cells	Form a lumenized tube growing behind the tip cells, proliferate, elongate the sprouts and construct blood circulation under suitable conditions.
Phalanx cells	Emerge in the final step during angiogenesis by lining vessels once the new vessel branches have been consolidated and also are engaged in optimizing blood flow, tissue perfusion, and oxygenation
Mural cells: pericytes and smooth muscle cells	Contribute to vessel lumen formation by strong cell-cell adhesion and tight junctions, are embedded in a thick basement membrane, and remain attached.

The balance between pro-angiogenic factors (especially the ones involved in NOTCH pathway) is implicated in the reversibility of transiently specification as tip or stalk cells ECs. In the endothelial tip, cells were observed with low NOTCH signaling activity levels and high levels of expression of DLL4 and VEGFR2 during sprouting (Gerhardt et al., 2003). The increase of motility and sprout formation is due to EphrinB2, located in tip cells’ filopodia, where it increases endocytosis and activates VEGF-R2 and VEGF-R3, without affecting endothelial proliferation (Sawamiphak et al., 2010). The EphrinB2 receptor, EphB4 is not present at the tip cells level, but instead, it is expressed on ECs after the vessel’s growth, being found predominantly in large blood vessels. Its overexpression suppresses sprouting and switch vascular growth to circumferential enlargement (Erber et al., 2006).

In contrast with tip cells which express NOTCH ligand DLL4 for sprout guidance, stalk cells express lower DLL4, but with high NOTCH signaling activity by downregulating VEGFR2, VEGFR3, and neuropilin 1 (NRP1) concomitant with up-regulating VEGFR1 (del Toro et al., 2010). The NOTCH ligand JAGGED1 (JAG1) is expressed primarily by stalk cells being involved in activating NOTCH 1 and maintaining differential NOTCH activity by antagonizing DLL4 that signals back to tip cells (Potente et al., 2011). JAG1- NOTCH signaling pathways promote the sprouting process, unlike DLL4- NOTCH signaling, which inhibits angiogenic sprouting (Bridges et al., 2011).

As the master regulator of angiogenesis, the VEGF pathway comprehensively directs the process of angiogenesis, comprising endothelial cell sprouting, lumen development, vessel enlargement and permeability, and the microarchitecture of vascular networks (Ribatti and Crivellato, 2012). During vascular sprouting, VEGF induces endothelial cell polarization and determination of tip cell formation (Gerhardt et al., 2003). At the same time, adjacent cells to stalk cells are converted by NOTCH signaling leading to VEGF receptor expression (Hellström et al., 2007; Potente et al., 2011).

At the vascular front, ECs’ phenotypic specialization as tip or stalk cells is defined as a transient and reversible process determined by the equilibrium between pro-angiogenic factors. The high level VEGF exposure of ECs selects them to become tip cells and regulates migration outward from the parent vessel up the gradient (Ribatti and Crivellato, 2012). In tip cells, VEGF induces the formation and extension of filopodia abundant in VEGFR-2. Filopodia guides the migration of the sprout in the nearby tissue to form a “bond” with other tip or stalk cells, moving toward each other and developing a new vessel (Figures 2B–D; De Smet et al., 2009). In a brief description, in response to a VEGF gradient, tip cells up-regulate the expression of ligand DLL4, leading to activation of NOTCH in stalk cells which further inhibit VEGFR-2 expression. Therefore stalk cells become less responsive to VEGF (that regulates proliferation of stalk cells), thereby ensuring tip cell activation (Adair and Montani, 2010). Concomitantly, VEGFR2 signaling up-regulates GLUT1 and PFKFB3 (6-Phosphofructo-2-Kinase/Fructose-2,6-Biphosphatase 3), thus increasing filopodia activity in tip cells (Fitzgerald et al., 2018). NOTCH inactivation is associated with disruption of the vascular hierarchy because the tip cells are not equipped to strengthen stable junctional complexes, explaining why inhibition of NOTCH in tumors results in suppressing tumor growth despite a high number of ECs and sprouts (Adair and Montani, 2010). NOTCH activation consists of proteolytic cleavage of NOTCH receptors and release of the intracellular domain (NICD). The NICD is translocated to the nucleus and interacts with the transcription factor CBF1 and Mastermind-like proteins leading to the activation of target gene expression and p300 histone acetyltransferases to turn on the expression of NOTCH target genes (Phng and Gerhardt, 2009). Acetylation on lysines of NCID enhances NOTCH activity by interfering with turnover of NICD, while associated SIRT1- NICD functions as a deacetylase that opposes NICD stabilization, thus restricting NOTCH activity (Guarani et al., 2011).

Endothelial tip cell filopodia are projected against the direction of interstitial flow or in the direction of an increasing VEGF gradient (Song and Munn, 2011). ECs equipped with guidance receptors at the filopodia level, which determines the back and forth movement for migration of ECs. For instance, ROBO4 is actively involved in filopodia constitution by remodeling and regulating the actin cytoskeleton (Sheldon et al., 2009). Also, ROBO4 maintains vessel integrity by activating and determining filopodia retraction of ECs (Larrivée et al., 2001). Similarly, PLEXIN-D1 is critical in limiting tip cells by modulating the ratio between tip and stalk cells in response to the VEGF gradient and modulating filopodia’s retraction. NRP and EPH family members are also involved in sprouting dynamics managing bidirectional signaling in tip cells by expressing the receptor or the ligand (Sawamiphak et al., 2010).

At the commencement of sprouting, a branch point in the vessel wall is produced by the degradation of BM and ECM from nearby capillaries. The structural alteration of the BM on the side of the enlarged mother vessel placed close to the angiogenic stimulus allows pro-angiogenic factors to discharge from the ECM (Tonini et al., 2003). ECs junctions become altered, and the space created in the matrix is invaded by ECs that initiate migration and proliferate within the somatic or tumor mass (Theocharis et al., 2016). ECM is the space between the tissue and vessels containing interstitial fluid, collagen, elastin, fibronectin, laminins, several other glycoproteins, and connective tissue cells (macrophages, fibroblasts, and plasma cells (Theocharis et al., 2016). Very low or high values of matrix densities (due to contents in collagen, elastin, etc.) will not allow the migration and proliferation of ECs only with an intermediate optimal matrix density (Bazmara et al., 2015). As heterodimeric receptors involved in adhesion to the ECM, integrins play a key role in regulating ECs’ sprouting ability by determining whether they can survive and adhere to a particular microenvironment (Humphries et al., 2006). Integrins control the adhesion and migration of ECs, as well as the proliferation and cells survival. The integrins, αvβ3 and αvβ5 are involved in promoting distinct pathways of angiogenesis: αvβ3 is required to initiate vessel growing through basic fibroblast growth factor (bFGF) or tumor necrosis factor α (TNF-α) and αvβ5 integrins by VEGF or transforming growth factor α (TGF-α) signaling pathways (see Box 2) (Weis and Cheresh, 2011).

**Box 2. Fibroblast Growth Factor (FGF).**

FGF, an angiogenic growth factor as heparin-binding protein, has a strong stimulatory effect in endothelial cell migration and proliferation. This is placed in the vascular BM and is up-regulated in the course of active angiogenesis. The most common fibroblast growth factors are FGF-2 or basic FGF (bFGF) and FGF-1 or acidic FGF (aFGF), which binds to their complementary receptors FGFR-1 or FGFR-2 (Gerwins et al., 2000). FGF2 stimulates angiogenesis by directly binding to FGFR1, increasing EC migration, promoting capillary morphogenesis (Presta et al., 2005). Also, FGF-2 up-regulate the expression of matrix metalloproteases (MMPs) and urokinase-type plasminogen activator (uPA) and stimulate degradation of the ECM and the stimulation of collagen, fibronectin and proteoglycans synthesis by ECs (see Box 3) (Lu et al., 2011). The lack of FGF2 determines integrity defects in ECs, required for sustaining endothelial cell proliferation and vessels restoration in damaged tissues. Also, FGF5 stimulate blood flow and enhanced vessel formation into the injured myocardium. FGF2 is critical for inducing angiogenesis in the presence of VEGF-A, treatment of ECs with FGF2 inhibitors causes the formation of vessels with abnormal architecture, including prominent filopodia suggesting that FGF2 has an important role in vascular integrity during angiogenesis (Seo et al., 2016).

Box 3 Tumor cells and the supporting cells secrete matrix metalloproteinases (MMPs) that can modulate angiogenesis by several mechanisms through direct cleavage of cell surface molecules or by exposing cryptic cell-binding sites in the ECM (Deryugina and Quigley, 2015). MMPs release pro-angiogenic growth factors that are sequestered in the matrix, such as VEGF and FGF and generate anti-angiogenic molecules (such as tumstatin and angiostatin) by dividing plasma proteins, matrix molecules, or proteases themselves to inhibit the angiogenesis process or coordinate branching (Arroyo and Iruela-Arispe, 2010). The inhibition of MMP functions is performed by tissue inhibitors of the matrix metalloproteinase (TIMPs) secreted by tumor or somatic cells. TIMP-1 binds to MMP-9 and inhibits its active form by binding to the zymogen forms of the enzyme (Deryugina and Quigley, 2015).

**Box 3. MMP family members roles in angiogenesis.**

MMP family members, zinc-dependent endopeptidases, can be organized into several groups, characterized by different substrate specificities such as gelatinases, collagenases, stromelysins, matrilysins and membrane-type MMPs (Nagase et al., 2006). MMP1 (a collagenase, enriched in tip cells) and MMP2 (a gelatinase) are expressed during angiogenesis and act to degrade extracellular matrix components. MMP-9 plays an important role in cancer cell invasion and tumor metastases (Hao, 2015). MMP-9 acts directly by degrading ECM proteins and activates cytokines and chemokines to regulate tissue remodeling in a large spectrum of physiologic and pathophysiologic processes (Yabluchanskiy et al., 2013). MMP-9 protein contains a catalytic domain that contains fibronectin type II domains, essential for substrate binding and degradation (Vandooren et al., 2013). MMP-9 interacts with substrates with hemopexin-like domain required for specific substrate recognition. ECM degradation initiated by MMP9 is succeeded by activation of VEGF and FGF-2. Also, in matrix remodeling and tumors stromal invasion MMP9 is increased by EGF/EGFR signaling (Deryugina and Quigley, 2015). MMP-9 stimulates macrophages, neutrophils and mast cells to initiate angiogenesis and, therefore, pathogenesis and intensify disease progression by MMP9-mediated activation of VEGF (Huang, 2018).

### Patterning of Vascular Networks and Vessel Maturation

After the migration of ECs into the surrounding matrix, the angiogenesis process continues with the re-organization of newly formed ECs to form tubules and create a new basement membrane for vascular stability (Tonini et al., 2003). New vessel circuits ascend when a tip cell contact other tip cells, and the BM is constantly deposited by the polarized ECs. Single sprouts headed by tip cells extending long filopodia direct the migration of the sprout in the nearby tissue. After forming anastomosis and blood flow in the capillaries, it starts to create shear stress, decreasing the VEGF-induced activity of ECs (Bazmara et al., 2015). Once vascularization of the tissue is reestablished, the pro-angiogenic factors are down-regulated, and ECs institute a quiescent phenotype, stable and highly interconnected with strong vessels walls, secreting VE-cadherins (Potente et al., 2011). Vascular maturation is induced by blood perfusion that determines pericyte recruitment and maturation and BM deposition (Figures 2E,F; Potente et al., 2011). However, loop capillary survival, elongation, and stabilization depend on blood flow pressure and changes in capillary diameter. Uncontrolled capillary blood flow and shear stress will determine the collapse of new loop formation (Bazmara et al., 2015). Pericytes covering the vessels support newly developed vessel structures and prevent their collapse, allowing the lumen to accommodate the size and volume of tumor cell aggregates and blood flow (Goel et al., 2011). This is a necessary ability of tumor angiogenic vasculature to sustain metastasis (Raza et al., 2010).

An essential feature for the proper function of vessels is maturation and coverage by mural cells (pericyte and vascular smooth muscle cells). Some growth factors, such as PDGFs, angiopoietins and TGF-β, contribute to this process. In capillaries and newly formed vessels, pericytes create direct contact with ECs, whereas arteries and veins are consolidated by layers of smooth muscle cells and a matrix (Bergers and Song, 2005). The interaction between ECs and pericytes for the vessel’s consolidation and stabilization involves the angiopoietin–Tie2 signaling pathway. Pericytes migrate and proliferate on the sprouting site and release TGFβ and angiopoietin-1 in a paracrine manner to suppress ECs proliferation and lead to endothelial layer maturation (Bergers and Song, 2005). TGF-β has considerable angiogenesis activities, starting with coordinating adjustment from vascular inhibition to pro-angiogenic activity depending on the context (Ferrari et al., 2012). It was noted that maintaining an endothelial quiescence state by TGF-β signaling through the TGFBR1/ALK5 receptor inhibits angiogenesis initiation while signaling through the ACVRL1/ALK1 receptor stimulates EC proliferation and migration (Gaengel et al., 2009).

### Survival and Vessel Perfusion

Blood flow and fluid shear stress strongly inhibit cell apoptosis and act as survival indicators for ECs (Rostama et al., 2014). Shear stress and pressure are essential to coordinate vessel diameters along with blood flow (Pries et al., 2010). Newly formed vessels regulate their form to fulfill oxygen demands from tissues by activating HIFs (Hypoxia Inducible Factor) that respond to changes in oxygen tension in ECs. HIF activity is regulated by prolyl hydroxylase domain proteins (PHD), which can sense the concentration of oxygen (Fan et al., 2014). Under normoxia, PHDs bind the oxygen molecules to hydroxylate HIFs, and hydroxylated HIF is recognized by the ubiquitin E3 ligase, and eliminated by proteasomal degradation. In hypoxic conditions, oxygen sensors become inactive and HIFs escape from degradation, leading to increased soluble VEGFR1 and VE-cadherin levels, thus allowing ECs to readjust vessel shape to their function of oxygen delivery dynamically. Particularly, in hypoxic conditions, oxygen sensors regulate in a feedback loop the expression of VE-cadherin to optimize vessel perfusions due to insufficient oxygen supply (Mazzone et al., 2009).

During maturation, the EC maintains the integrity of the microvessels lining and protects the vessel wall from environmental stresses by autocrine and paracrine survival signals. Differentiated pericytes produce VEGF as a survival factor for EC in vessels, so that inhibition of VEGF induces apoptosis in endothelial and mesenchymal cell cocultures (Darland et al., 2003). VEGF activates the PI3K/AKT survival pathway of ECs in physiological conditions. VEGF activity also has a paracrine activity in angiogenesis stimulation, such that inhibition of VEGF in ECs does not affect vascular development (Duffy et al., 2000). Endothelial cell communication with vascular smooth muscle cells is mediated via NOTCH signaling on adjacent cells. NOTCH signaling sustains vascular homeostasis due to its ability to establish mature vessels that promote perfusion and relieve tissue hypoxia. Mural cells promote vessel stability through NOTCH – DLL4 signaling by sustaining the accumulation of BM components (Rostama et al., 2014).

### Vascular Modeling in Angiogenesis

Modeling and development of blood vessels is a multi-step process that involves different mechanisms of angiogenesis. The initial stages of vascular growth in expanding tissue are mainly promoted by sprouting angiogenesis. Remodeling of the vascular network is performed by splitting angiogenesis, described as intussusceptive angiogenesis, vascular cooption and vascular mimicry (De Spiegelaere et al., 2012).

#### Intussusceptive Angiogenesis (IA)

Intussusceptive microvascular growth is a fast process (hours or even minutes) that occurs without ECs proliferation and without interfering with the local physiological conditions with relatively lower metabolic cost (Figure 1D). As an intravascular growth mechanism, vessel intussusception involves the division of pre-existing vessels into two new vasoformations (De Spiegelaere et al., 2012; Zuazo-Gaztelu and Casanovas, 2018), remodeling through vascular reduction and increased tissue volume, developing the capillary network without a high metabolic demand (Phng and Gerhardt, 2009). This process requires the interaction between two ECs on the opposite walls that form a cell bridge, based on the endothelial bilayer with cell-cell junctions and the interstitial pillar. The process includes the recruitment of pericytes and mural cells that cover the wall, which is further enlarged, allowing the retraction of ECs and developing two connected but separate vessels (Zuazo-Gaztelu and Casanovas, 2018).). The degree of vascular enlargement is proportional to VEGF dose. Both low and high VEGF expression initiates intraluminal pillar formation, but excessive diameters lead to the failure of the formation of a normal vasculature, leading to the failure of division and atypical blood vessels separated by collagen fibers (Ozawa et al., 2004). In order to stabilize nascent vascular structures, pericyte recruitment is crucial. Loss of pericytes layers by incomplete angiogenesis in adult tissue leads to persistent proliferation of ECs and increased fragile vascular structures (Banfi et al., 2012). The molecular mechanism of the switch among normal and abnormal angiogenesis by VEGF dose is associated with the EphrinB2-EphB4 pathway. Specific VEGF doses regulate EphB4 to control endothelial proliferation, size of the initial vascular enlargement, and modulating its downstream signaling through MAPK/ERK. While EphrinB2 sustain tip cell migration in sprouting by the VEGF pathway, EphB4 diminishes endothelial proliferation during intussusceptive angiogenesis by VEGF-induced ERK1/2 phosphorylation (Groppa et al., 2018).

#### Vascular Cooption in Tumor Tissue

Some highly vascularized primary and metastatic tumors use pre-existing host tissue vessels as their blood supply by expressing a mixed phenotype with co-opted vessels and angiogenesis (Figure 1B). Also, they can grow to an assured level without causing a specific angiogenic response (Donnem et al., 2013). Faster as an angiogenic response by sprouting, tumors connect with the host vessels in the stroma and with increasing size of cellular mass, the blood vessels become completely embedded in the tumor (Küsters et al., 2002). Co-opted blood vessels are commonly detected in highly vascularized tissue such as the brain, lung, liver, and lymph nodes (Donnem et al., 2013; Kuczynski et al., 2019). The key players for ECs survival during cooption are VEGF and ANG-1, which supports tumor vessel maintenance. Shortly after that, tumor coopted vessels begin to fail as a host defense mechanism by overexpressing ANG-2 that disrupts the interaction between ANG-1 and Tie-2 and causes destabilization of capillary walls (Holash et al., 1999). Vessel cooption is implicated in patient outcomes and resistance to cancer therapies and is a valid target of new therapeutic strategies (Kuczynski et al., 2019). In some cases, it was observed that anti-angiogenic treatment is less operative in the tumor periphery, meaning that vessel cooption is located at the edge of tumors (Donnem et al., 2013). Oder tumors with a profound central devascularized upon sunitinib treatment continued to be well vascularized at the upper edge of these aggressive tumors (Welti et al., 2012).

#### Vasculogenic Mimicry (VM) in Tumor Tissue

This mechanism refers to the tumor cells that adopt an endothelial phenotype and determine *de novo* development of the matrix infused vasculogenic-like system as alternative mechanisms for re-vascularization (Figure 1C; De Spiegelaere et al., 2012). The vascular mimicry process results in a capillary network completely composed of tumor cells or mosaic vessels alternating tumoral cells and ECs in the vascular walls (Angara et al., 2017). It reflects the plasticity of aggressive behavior and easily metastasizes toward distant sites, which express vascular cell markers and line the tumor vasculature. Therapeutic strategies that target ECs do not affect tumor cells that engage in VM (Zhang et al., 2007). The morphological, clinical and molecular characterization of vasculogenic mimicry has been described in almost all carcinogenic mass types and was linked to unfavorable outcomes of malignancies (Ge and Luo, 2018). Vasculogenic mimicry describes tumor cells’ ability to undergo epithelial-mesenchymal transition (EMT) and achieve cancer stem cells like phenotypes. Those cells are transdifferentiated into endothelial-like cells that support the ECM formation, which is CD31 negative and express PAS-positive patterned vasculogenic networks. The new networks are connected to the mother vessel but with distinct microvessels that provide suitable nutrient supply and contribute to tumor progression (Folberg et al., 2000). For example, in aggressive melanomas, the tumor cells adopt an endothelial-like phenotype characterized by overexpression of specific endothelial genes (Folberg et al., 2000) and meticulously reproduce vasculogenesis starting from dedifferentiated tumor cells. While VE-cadherin is considered specific for ECs, it has been detected in tumoral cells with aggressive phenotypes except for benign masses, and also its down-regulation is conductive to the loss of VM formation in melanoma (Ge and Luo, 2018). Usually, anti-angiogenic drugs target ECs by initiating endothelial cell apoptosis and proliferation inhibition to reduce tumor vascularization. The incidence of vascular mimicry may complicate common anti-angiogenic strategies to treat certain tumors, because the vasculature can be adjusted to another angiogenic phenotype (De Spiegelaere et al., 2012). Remaining malignant cells at a tumoral site can form VM channels, providing oxygen and nutrients that sustain cancer progression. On the other hand, resistance to anti-angiogenic therapies leads to cancer recurrence. Taken together, VM channels increase intratumoral therapeutic agent delivery but with a reduction of drug efficacy of conventional chemotherapeutic agents (Lezcano et al., 2014; Ge and Luo, 2018). Therefore, the development of novel chemotherapies is urgently needed to abolish VM structures and improve tumor therapy.

#### Endothelial Progenitor Cells Promote Angiogenesis

In some cases, endothelial progenitor cells or angioblasts that are a CD34-enriched subpopulation of mononuclear blood cells, are able to adapt to an adherent endothelial phenotype. This model supposes that new vessels can also grow by recruiting endothelial progenitor cells (EPCs) circulating in the blood (Asahara et al., 1997). Recruitment and integration of EPCs into angiogenesis is a coordinated process that includes chemoattraction and cell arrest in the vessel wall, interstitial migration and incorporation into the vasculature, and endothelial cell differentiation (Hillen and Griffioen, 2007). The recruitment of EPCs is produced under physiological trauma or stress and tumor growth. It starts with the activation of MMP9, detachment of the progenitor cells (c-kit positive) from the bone marrow niche and release of EPCs from the bone marrow into the circulation (Heissig et al., 2002). Angiogenic factors like PLGF, VEGF, MMP9, SDF1, ANG-1, selectin and integrins are essential for the active arrest of EPCs to the vasculature and transendothelial migration (Hillen and Griffioen, 2007). The EPCs undergo a change in specific markers from EPCs endothelial-like such as CD14, CD34, CD31, Tie-2 and VEGFR2 (Kalka et al., 2000) toward a mature endothelial cell pattern (VE-cadherin, von Willebrand factor and eNOS) (Hillen and Griffioen, 2007). However, the characterization of EPC subpopulations is yet to be fully elucidated, and the exact molecular pathways involved in the mobilization and recruiting of EPCs are complex and still under investigation.

## Angiogenesis in Disease Progression

In physiological angiogenesis, cells located more than 200 μm from a blood vessel determine new blood oxygen and nutrients source’s recruitment and remove waste products. Solid tumors development involves synchronization between the neovascularization process and tumor cells proliferation, a process regulated by endogenous activators and the angiogenic process’s inhibitor (Tonini et al., 2003). The progressive expansion of the tumor exceeds the capacity to support the existing vasculature and is limited to the tumor’s periphery, leading to low oxygenation (hypoxia) and tumor progression (Krock et al., 2011; Dulloo et al., 2015).

Hypoxia induces pro-angiogenic factors in excess, creating a local imbalance that leads to new blood vessels recruitment. These can be poorly organized, fragile, with low functionality, causing low oxygen areas in the tumor that generate a persistent angiogenic signal (Vaupel et al., 2001; Krock et al., 2011). Initially, the hypoxic condition promotes cell death in the tumor mass, but in this way, it provides constant angiogenic signals, allowing survival in the absence of oxygen. In these hypoxic microregions, the resistant cells are selected based on their ability to survive and contribute to a potent tumor mass with very malignant characteristics (Wouters et al., 2003). HIF-1, the oxygen-sensitive transcription factor, acts as heterodimer containing β subunit that is constitutively expressed and one of three alpha subunits (HIF-1α, HIF-2α, or HIF-3α). HIF activity is regulated mainly by post-translational changes in various amino acid residues of its alpha subunits. Both β and α subunits belong to the group of the basic helix-loop-helix (bHLH)-PER-ARNT-SIM (PAS) protein class (Zhang et al., 2011). HIF function is mainly regulated by a stabilized HIF-1α subunit, which is translocated to the nucleus upon hypoxic induction, where it dimerizes with HIF-1β via bHLH and PAS motifs. The activity of HIF-1 is controlled mainly by regulating protein levels at various amino acid residues of the α subunit (Lee et al., 2004). The HIF-1α subunit becomes stable and recruits co-activators such as p300/CBP and HIF-1β to control its transcriptional activity and change transcriptional levels of a hypoxia-specific miR-210 (Wang et al., 2007). Thus HIF-1α is tightly regulated to protect itself against degradation by the tumor suppressor von Hippel-Lindau in hypoxic conditions (Lee et al., 2004). Moreover, HIF-1 is a master modulator for hypoxic gene expression and signaling that can simultaneously induce the transcriptional expression of both proangiogenic factors, VEGF and MMP-9 in a coordinated fashion (Deryugina and Quigley, 2015). The high expression of HIF-1α level in tumor tissues can up-regulate VEGF expression and determine loss of E-cadherin expression, thus reducing cell-cell adhesion and facilitating tumor cell metastasis (Vaupel et al., 2001).

### Pro- and Anti-angiogenic Factors in Tumor Angiogenesis

A balance between angiogenic inductors and inhibitors results in quiescent vasculature in normal adult tissue. Similarly, angiogenesis is dormant in small tumors that start as avascular masses, depending on their microenvironment vasculature (Baeriswyl and Christofori, 2009). Disturbance of balance in physiological angiogenesis generates wound healing, but in pathological angiogenesis, it results in an outgrowing vascularized tumor and eventually to malignant tumor progression (Huang and Bao, 2003). In addition to vessel growth, HIF1 is induced as an essential gene expression regulator, when tumor tissues exceed the oxygen diffusion limit (Dulloo et al., 2015) and induce pro-angiogenic compounds targeting tumor vascular supply, disturbing the existing vessels (Table 2). These facts activate the “angiogenic switch,” which sustains tumor growth and metastasis process (Huang and Bao, 2003). Therefore, hypoxic tumor cells around the necrotic nucleus overexpress VEGF, which controls the formation of new blood vessels from the existing normal vasculature adjacent to the hypoxic site. The onset of angiogenesis involves both endothelial cell proliferation and increased vascular permeability. Endothelial progenitor cells derived from bone marrow or mesenchymal or hematopoietic stem cells that migrate from the systemic circulation to tumors are also recruited to form blood vessels (Das and Marsden, 2013).

**TABLE 2:** Pro- and antiangiogenic their importance in the angiogenesis process.

Angiogenic factors	Major function in angiogenesis process	References
**Pro-angiogenic factors**		
VEGF-A, PlGF, VEGF-B, VEGF-C, VEGF-D, VEGF-E	Vasculature formation; Formation of primitive endothelial tubes; Induce proliferation, migration, and differentiation of EC	Kazemi et al., 2002
aFGF, bFGF	Development and maintenance of a mature vascular network	Eguchi et al., 1992; Lee et al., 2000
MMP-9; MMP-14	Direct degradation of ECM proteins; overexpression of MMP-14 increases VEGF production and angiogenesis in glioblastomas	Deryugina and Quigley, 2015
Angiogenin	Support endothelial survival and proliferation, endothelial tube development	Miyake et al., 2015
FGF2, FGF5	Induce cell sprouting elongated morphology with prominent filopodia; endothelial cell growth and movement	Seo et al., 2016
TGFα/β, PDGF, TNFα, HGF/SF, COX-2	Induce differentiation of EC, proliferation and migration	Yoo and Kwon, 2013
NPAS4	Regulates VE-cadherin expression and regulates sprouting angiogenesis and tip cell formation	Esser et al., 2015
TBX3	Its overexpression drives angiogenesis and tumor expansion *in vivo*	Krstic et al., 2020
Nuclear factor 90	Sustain tube formation and cell migration of HUVECs and expression of VEGF-A induced by hypoxia in cancer	Zhang et al., 2018
TSLP	Promotes angiogenesis in cervical cancer	Zhou et al., 2017
TXNDC5	Down-regulate SERPINF1 and TRAF1 expression; sustain atypical angiogenesis, vasculogenic mimicry and metastasis	Xu et al., 2017
IL-1, IL-5, IL-6, IL-8, IL-17	Pro-inflammatory chemokines secreted by leukocytes and macrophages. Induce endothelial cell tube formation and vascularization	Ribatti, 2018
LAPTM4B	Promote tumor angiogenesis through the HIF-1α and VEGF pathway	Meng et al., 2015
CD146	New coreceptor for VEGFR-2; EMT inducer; promotes endothelial cell migration and angiogenesis and human tumor growth	Jiang et al., 2012
**Anti-angiogenic factors**		
Angiopoietin: ANG-1 ANG-2	Stabilization of existing vessels, control the interaction between ECs and the surrounding environment; ANG-1 suppresses ICAM-1, VCAM-1 and E-selectin; ANG-2 is an antagonist for ANG-1 and inhibits blood vessels maturation	Huang and Bao, 2003
TSP-1, TSP-2	Suppresses migration and induces endothelial cell apoptosis by blocking angiogenic factor access to co-receptors on the endothelial cell surface	Huang and Bao, 2003
TIMP1	Inhibit MMP9 or uPA activity; binds to MMP-9 to inhibit its active form	Yabluchanskiy et al., 2013
Angiostatin	Directly inhibit neutrophil migration and neutrophil-mediated angiogenesis	Benelli et al., 2002
Endostatin	Inhibition of ECs proliferation and migration, tube formation	Ergün et al., 2010
Vasostatin	Angiogenesis inhibitor that specifically targets proliferating ECs	Pike et al., 1998
Interferon-alpha	IFN α – decrease VEGF gene expression	von Marschall et al., 2003
Interleukins: IL-1β, IL-4, IL-10, IL-12, IL-18, IL-23, IL-25, IL-27	Inhibits endothelial cell proliferation	Ribatti, 2018
TGF -beta	Suppresses VEGFA-mediated angiogenesis and tumor progression and metastasis	Geng et al., 2013
SEMA3A	Endogenous inhibitor expressed in premalignant lesions that disappear during tumor progression.	Maione et al., 2009

*Cox-2, cyclooxygenase-2; PlGF, placental growth factor; NPAS4, neuronal PAS domain protein 4; TBX3, T-box transcription factor 3; HGF/SF, hepatocyte growth factor/scatter factor; TSLP, thymic stromal lymphopoietin; TXNDC5, thioredoxin domain-containing protein 5; CD146, cluster of differentiation 146; ANG, angiopoietin; ICAM-1, intercellular adhesion molecule-1; VCAM-1, vascular cell adhesion molecule-1; TSP, thrombospondin; TIMP1, TIMP metallopeptidase inhibitor 1; SEMA3A, semaphorin 3A.*

In general, tumor blood vessels are considered leaky and inefficient in oxygenation, raising hypoxia, including the generation of growth factors and cytokines by the surrounding tumor cells. This, in turn, promotes tumor invasion and metastasis. Therefore, the concept of vessel “normalization” has gained significant attention in order to control the tumor vasculature to improve drug delivery (Warren and Iruela-Arispe, 2010).

### Tumors Micro-Environment

The tumor microenvironment consists of a mixture of cells (tumoral cells, ECs, immune cells, fibroblasts) and extracellular matrix surrounding or infiltrating tumor tissues. Meanwhile, tumor stroma can produce factors to acquire blood vessels for proper supplies of oxygen, nutrients, and waste disposal. Therefore tumoral angiogenesis is considered to be essential for tumor progression and metastatic dissemination (Bhome et al., 2015). The tumor microenvironment comprises several pro-angiogenic factors, including VEGF, bFGF, PDGF, MMP and inflammatory cytokines secreted by tumor cells or tumor-infiltrating lymphocytes or macrophages. These tumoral cells may increase a pre-existing invasion program activity, activate pro-angiogenic gene expression, and stimulate angiogenesis, ensuring tumor cell motility, invasion, and metastasis (Goel et al., 2011).

## Epigenetic Control of Angiogenesis in Tumor Development

The suppression of tumor suppressor genes or the activation of oncogenes induced by tumor development and progression is associated with reversible epigenetic dysregulation. Global alteration of epigenetic modification is considered one of the hallmarks of cancer. While it is known that genetic changes (DNA mutations, insertions, deletions, chromosomal rearrangement) have an essential role in the altering of target genes in cancer cells, the importance of epigenetic mechanisms becomes an important event in the early development and late stages of tumor development. Epigenetic modification of tumor cells includes diverse reinforcing and converging signals, including DNA methylation, covalent modifications of histones, nucleosome- DNA interactions and small inhibitory RNA molecules.

### DNA Methylation of Anti-angiogenic Factors

Angiogenesis initiation marks the conversion process from a dormant cellular state to active growth of ECs, a common disease progression feature. DNA methylation occurs as modification of cytosines nucleotides placed within CpG dinucleotides. Methylation abnormalities commonly occur in many pathological disorders in the form of hypermethylation of CpGs within promoter regions of genes, and therefore these tumor suppressors are silenced. In contrast, global genomic hypomethylation or demethylation has been found in many types of tumors and is associated with activation of proto-oncogenes and generation of chromosomal instability (Hellebrekers et al., 2007).

The most common epigenetic changes in tumoral cells include hypermethylation of anti-angiogenic factors, a mechanism of the tumor to promote angiogenic pathways. For example, methylation-induced silencing gene expression of the angiogenesis inhibitor thrombospondin 1 (THBS-1) inhibits the secretion of TGFß, which was correlated with increased metastasis, invasion, and poor prognosis in several types of cancers (Buysschaert et al., 2008). Also, hypermethylation of proteases with anti-angiogenic properties, such as ADAMTS-8, tissue inhibitor of MMP2 and MMP3 (TIMP-2 and –3), was observed in various tumors (Hellebrekers et al., 2007).

Vascular endothelial growth factor, its receptors and eNOS, essential genes critical for angiogenesis promotion, also are controlled by their gene promoter methylation status. VEGF and eNOS expressions were downregulated via Methyl-CpG–Binding Domain Protein 2 (MBD2) binding, the protein readers of methylation that regulate endothelial function in both physiological and disease states (Rao et al., 2011). In physiological conditions, ECs expressed moderate levels of MBD2, but the lack of MBD2 significantly enriched angiogenesis and secured ECs from H_2_O_2_-induced apoptosis. Reduced expression of MBD2 determines a higher ERK1/2 activity in ECs, which promotes apoptosis by increasing the expression of BCL-2. In ischemic insults, the essential endothelial genes such as eNOS and VEGFR 2 undergo a DNA methylation turnover, and MBD2 reads DNA methylation changes signals for mediating gene silencing (Rao et al., 2011). MBD2 overexpression has been detected in various solid tumors and promotes cancer progression by mediating silencing of tumor suppressor genes (Gong et al., 2020). MBD2 binds to the gene promoter of brain angiogenesis inhibitor 1 (BAI1) and inhibits its antiangiogenic and antitumorigenic properties in glioma cells. 5-Aza-dC treatment released MBD2 led to reactivation of functional activity of BAI1 *in vitro* and *in vivo* (Zhu et al., 2011). However, MBD2 has dynamic activity and functions in a cell, and tissue-specific way and its expression is associated with malignancy and poor prognosis in solid tumors. In lung cancer, reduced expression of MBD2 was associated with disease progression metastasis and severe patient prognosis (Pei et al., 2019). MBD2 is component of Mi-2/NuRD (Nucleosome Remodeling Deacetylase) complex which reveals nucleosome remodeling and histone deacetylation activities. MBD2/NuRD complex deacetylates the nucleosomes surrounding the targeting site. MBD2 and MBD3 appear to have both activating and repressive roles when complexed with NuRD/Mi-2 in a context-dependent manner (Menafra and Stunnenberg, 2014).

### Histone Modifications Defining Angiogenesis

Eukaryotic DNA is tightly packaged into chromosomes around histone protein complexes, forming nucleosomes folded into chromatin structures, and for gene regulation, the histone amino (N)-terminal tails extending from the nucleosomal cores are acetylated and deacetylated at ε-acetyllysine residues by histone acetyltransferases (HATs) and histone deacetylases (HDACs) respectively. HATs sustain gene transcription through acetylation of histones, influencing the charge neutralization and relaxing nucleosomes structure, and HDACs modulate chromatin structure by promoting histones’ deacetylation and suppressing transcription by condensing the chromatin (Sasaki and Matsui, 2008).

Histone deacetylases are involved in maintaining vascular integrity due to the association of endothelial growth and vascular morphogenesis with increases in HDAC1 activity and its export from the nucleus (Table 3). Also, inhibition of HDAC1 diminishes endothelial morphogenesis and MMP14 gene expression, potentially through its role as a transcriptional regulator (Bazou et al., 2016). HDAC1 also regulates VEGF expression in normal keratinocytes (Reynoso-Roldan et al., 2012). It was shown that HDAC1 supplements and amplifies the VEGF signaling pathway and activate angiogenesis when interstitial flow signals are existent in angiogenic sprouting (Bazou et al., 2016). Overexpression of HDAC1 inhibits p53 and von Hippel-Lindau tumor suppressor gene expression and promotes angiogenesis under hypoxic conditions (Kim et al., 2001).

**TABLE 3:** The enzymes that modify histones in angiogenesis.

Enzyme	Activity	Target	References
G9a	Histone methyltransferase	Histone H3 lysine 9 (H3-K9) methylation	Pirola et al., 2018
DOT1L	Histone methyltransferase	H3K79 methylation, H3K9 dimethylation and H4K20 tri-methylation	Jones et al., 2008
SET7	Histone methyltransferase	Methylates H3K4	Zhang et al., 2016
JMJD1A	Histone demethylases	Demethylates Histone 3 lysine 9 (H3K9)	Osawa et al., 2013
LSD1	Histone demethylases	H3k4me1/2, h3k9me1/2	Perillo et al., 2020

*DOT1, like histone lysine methyltransferase; JMJD1A, jumonji domain containing 1A; LSD1, lysine-specific demethylase 1A.*

#### G9a

G9a, also known as EHMT2 (Euchromatic histone-lysine N-methyltransferase 2) a nuclear lysine histone methyltransferase that mainly catalyzes histone H3 lysine 9 (H3K9), is involved in cancer invasion and metastasis. Generally, G9a involves reversible modification of transcriptional gene silencing, but its activity is associated with tumoral angiogenesis and poor patient outcome (Pirola et al., 2018). A higher expression of G9a in aggressive cervical cancer than in normal epithelium cells was correlated with increased angiogenesis in cervical cancer (Chen et al., 2017). Inhibition of G9a histone methyltransferase significantly inhibits angiogenic factors like VEGF, interleukin-8 and angiogenin (Chen et al., 2017). In tumor angiogenesis, increased H3K9 acetylation was related to over-expression of TIMP3 inhibiting growth of carcinoma cells, while decreased H3K27me3 resulted in the upregulation of TIMP3 and re-expression of anti-angiogenic genes in dormant cells. In addition, an increase in both H3K4me3 and H3K9 was associated with higher CDH1 expression during dormancy. TIMP3 and CDH1 are overexpressed during the dormancy of cancer cells having antiangiogenic properties and are inhibited in the course of the conversion to active development by epigenetic changes (Lyu et al., 2013).

#### DOT1L

DOT1L is an H3K79 histone methyltransferase studied in embryogenesis, hematopoiesis, cardiac function, cycle regulation, DNA damage response and leukemia (Nguyen and Zhang, 2011). Knockout of DOT1L results in embryonic death during organogenesis due to abnormal cardiac morphogenesis and angiogenesis defects in the yolk sac. It plays an important role in heterochromatin formation, as DOT1L-deficient stem cells have low levels of H3K9 dimethylation and H4K20 tri-methylation in centromeres and telomeres and also show an overall loss of H3K79 methylation (Jones et al., 2008). In human umbilical vein endothelial cells (HUVECs), silencing of DOT1L decreases ECs viability and migration, tube formation and sprout development, and cannot establish a functional vascular network. The pro-angiogenic role of DOT1L, was also supported by the observation that DOT1L work together with the ETS-1 proto-oncogene to activate VEGFR2 expression, which further activates the ERK1/2 and AKT signaling pathways in order to achieve angiogenesis (Duan et al., 2013).

#### SET7

SET7 is a histone methyltransferase that specifically methylates H3K4 in humans, involved in angiogenesis by cooperating with the transcription factor GATA1, which promotes transcriptional up-regulation of VEGF and angiogenesis initiation. SET7 inhibition in breast cancer showed decreased VEGF secretion *in vitro* and *in vivo*, leading to lower proliferation rates, migration and tube formation of HUVECs and also inhibition of breast cancer development in nude mice. Also, GATA1 and SET7 were upregulated and associated with poor prognosis in breast cancer samples (Zhang et al., 2016). On the other hand, SET7/9 is highly expressed in breast tumoral tissues and cancer cell lines. Moreover, a recent study confirms the association between unfavorable prognosis in breast cancer with upregulated SET7/9 expression, describing a carcinogenicity mechanism of SET7/9 through activation of Runt-related transcription factor 2 (RUNX2). The same study showed that SET7/9 is negatively regulated by tripartite motif-containing protein 21 (TRIM21) through a proteasome-dependent mechanism and increased ubiquitination (Si et al., 2020).

#### Enhancer of Zeste Homolog 2

Enhancer of zeste homolog 2 (EZH2) promotes angiogenesis in a paracrine manner by methylating and silencing vasohibin1 (VASH1) (Lu et al., 2010). High EZH2 expression and low VASH1 in intrahepatic cholangiocarcinoma were associated with tumor angiogenesis initiation, poor disease-free survival, and poor overall survival (Nakagawa et al., 2018). Also, miR-101 was reported to target EZH2 in glioblastoma cells directly. *In vitro* and *in vivo* inhibition of EZH2 attenuated endothelial tubule formation, tumoral migration and invasion, resulting in generally reduced tumor growth (Smits et al., 2010).

#### JMJD1A

JMJD1A an epigenetic regulator that demethylates Histone 3 lysine 9 (H3K9), is expressed as a response to the cooperative action of hypoxia and nutrient starvation in cancer cells by promoting angiogenesis and macrophage infiltration. JMJD1A inhibition suppresses tumor growth and angiogenesis and sensitizes cancer cells to VEGF and VEGFR inhibitors by suppressing FGF2, HGF, and ANG2. Moreover, inhibition of JMJD1A sustained the antitumoral properties of two anti-angiogenic treatments (bevacizumab and sunitinib), limiting tumor resistance (Osawa et al., 2013).

#### LSD1

LSD1 remove methyl groups of H3K4me1/2, H3K9me1/2, and some non-histone substrates that mediate many cellular signaling pathways that are highly overexpressed in different types of cancers (Perillo et al., 2020). It also controls the turnover of HIF-1α as a cellular response to hypoxia. LSD1 promotes protein stability and tumor angiogenesis by demethylating the K391 residue and inhibiting HIF-1α downregulation with H_2_O_2_ production, which inhibits the hydroxylating activity of PHD_2_ on HIF-1α with its subsequent ubiquitination (Lee J. Y. et al., 2017).

### MicroRNAs (miRNAs) as Modulators in the Development of Angiogenesis

An essential role in regulating the angiogenesis process has been attributed to microRNAs. These are non-protein-coding RNA, small molecules with around 22 nucleotides. miRNAs regulate the expression of the target protein by two effects: *complete* matching the miRNA 5′ end with 3′ untranslated regions (3′-UTR) of target mRNA conduct to the reduction of targeted messenger mRNA and subsequently, their translation, or *incomplete* by repressing target protein translation without affecting mRNA stability (Schwerk and Savan, 2005).

MicroRNAs, generated from independent transcription units, can be either in polycistronic clusters or located within an intron of a protein-coding gene (Bartel, 2004; Suárez et al., 2008). The discovery of miRs that mediate post-transcriptional silencing of target genes has exposed exactly how non-coding RNAs play complex roles in angiogenesis. Initial evidence for the importance of miRs in regulating angiogenesis emerged from several experiments using a mutation in the Dicer gene, which is a ribonuclease essential for microRNA biogenesis (Kuehbacher et al., 2007; Suárez et al., 2008). EC-specific Dicer inactivation in mice reveals embryonic lethality and fails to determine a vasculogenic phenotype. With weakened angiogenic capacity and reduced endothelial tube development, the mouse embryo showed an alteration of proangiogenic factors genes and reduced angiogenic response (Yin et al., 2015). An increasing number of endothelial miRNAs have been reported to control the angiogenesis signaling pathways, thus controlling endothelial proliferation, migration and vascular integrity (Table 4; Yin et al., 2015).

**TABLE 4:** Pro-angiogenic and anti-angiogenic miRNAs and their role in the angiogenesis process.

microRNA	Suppression of target genes	Role in angiogenesis	References
**Pro angiogenic miRNAs**			
miR-222	c-kit, eNOS p27	Induce proliferation and cell cycle progression Inhibit p27 and increase cells clonogenicity	Fish et al., 2008; Ding et al., 2017
miR-17-5p, -18a, -19a, -20a, -19b, and -92a	TIMP1, TSP-1 PTEN, CTGF, TGFβ, SMAD3	Promotes ECs proliferation, survival and cord formation and angiogenesis Induce tumor angiogenesis, better-perfused tumors *in vivo* Supress c-Myc, which upregulate VEGF and downregulate TSP1 Direct repression of TSP-1 and CTGF	Knies-Bamforth et al., 2004; Fox et al., 2013
miR-424	VEGFA	Functions to promote VEGF signaling in glioma	Sun et al., 2020
miR-130a	MEOX2, HOXA5	Antagonizes the inhibitory effects of MEOX2 or HOXA5 on ECs tube formation	Chen and Gorski, 2008
miR-210	EphrinA3, HGF NPTX1, HOXA1, HOXA9, HOXA3, E3F3	Stimulates tube formation and migration of ECs in hypoxia Decrease proapoptotic signaling in a hypoxic tumor environment. VEGF-induced chemotaxis, HUVEC tubulogenesis and development of capillary-like structures	Fasanaro et al., 2008; Wang Z. et al., 2017; Fan et al., 2018
miR-296	HGF	Mediate tube formation and ECs migration and tumoral angiogenesis; Inhibit HGF which lead to VEGFR2 and PDGFR expression.	Würdinger et al., 2008
miR-378	Sufu, Fus-1	Promotes cell survival tumor growth and angiogenesis by reducing expression of tumor suppressor both Sufu and Fus-1	Lee et al., 2007
miR-126	SPRED1, VCAM1 and PIK3R2	Endothelial cell migration, re-organization of the cytoskeleton, capillary network stability and cell survival	Fish et al., 2008
miR-21	PTEN	Promote tumor angiogenesis by activating partially AKT and ERK and over-expression of HIF-1 and VEGF.	Liu et al., 2011
**Anti-angiogenic miRNAs**			
miR-221, 222	c-kit, eNOS, ZEB2	Reduces capilary tube formation, EC migration and angiogenesis	Celic et al., 2017
miR-15, miR-15b miR-16	VEGF, Bcl2	Induces apoptosis, cell cycle regulation and reduce tumor induced angiogenesis	Cimmino et al., 2005
miR-92a	Integrin α5	Inhibit VEGFA and integrin subunit alpha5	Bonauer et al., 2009
miR-27a/b	SEMA6A	Inhibit SEMA6A antiangiogenic activity, which controls the repulsion of adjacent ECs	Urbich et al., 2012
miRNA-29c	VEGF MMP2	Inhibits angiogenesis by downregulating VEGF and increase MMP-2 levels	Fan et al., 2013
miRNA-199a-3p	VEGFA, VEGFR1, VEGFR2, HGF MMP2	Overexpression suppressed cancer growth, angiogenesis and lung metastasis and HCC	Ghosh et al., 2017
miRNA-497 miR-29b	VEGFR2, VEGFA	Cell proliferation and migration during sprouting angiogenesis.	Rosano et al., 2020
miRNA-519c	HIF-1α, HIF-2α,	Inhibiting angiogenesis and metastasis of neuroblastoma	Cha et al., 2010
miR-543	ANG2	Inhibit angiogenesis in osteosarcoma	Wang L. H. et al., 2017
miRNA-9	MMP14, Stathmin	Inhibit vascular mimicry in glioma cell	Song et al., 2013
miRNA-181-5p	MMP14	Attenuating breast cancer cell migration, invasion and angiogenesis	Li et al., 2015
miRNA-135a	FAK	Inhibits ECs migration and invasion by targeting FAK pathway	Cheng et al., 2017
miRNA-218	ROBO1	Disturbed the tubular structure and inhibited the migration of ECs	Zhang et al., 2017
miR-9, miR-135a, miR-181a/b, miR-199b and miR-204	SIRT1	Downregulate SIRT1 and defective blood vessel formation	Saunders et al., 2010
miR-874	STAT3/VEGFA	Inhibit angiogenesis through STAT3/VEGFA pathway in gastric cancer	Zhang et al., 2015

*ZEB2, zinc finger E-Box binding homeobox2; PTEN, phosphatase and tensin homolog deleted on chromosome ten; CTGF, connective tissue growth factor; HGF, hepatocyte growth factor; SMAD3, SMAD family member 3; SPRED1, sprouty related EVH1 domain containing 1; PIK3R2, phosphoinositide-3-kinase regulatory subunit 2; HOXA5, homeobox A5; MEOX2, mesenchyme homeobox 2; NPTX1, neuronal pentraxin 1; FUS1, nuclear fusion protein; Robo1, Roundabout1; SIRT1, Sirtuins 1.*

Hypoxia-induced angiogenesis up-regulates the expressions of let-7 and miR-103/107 via activation of HIFs. These miRs suppress argonaute 1 (*AGO1*), which is required for the microRNA-induced silencing complex (miRISC) to silence VEGF mRNA, resulting in the translational de-suppression of VEGF (Chen et al., 2013). In ECs, miR-210 and other miRs regulated under hypoxic conditions directly down-modulate the expression of Ephrin-A3, a receptor protein-tyrosine kinase with effects on tubulogenesis and chemotaxis (Fasanaro et al., 2008). As well as proteins, there are some anti-and pro-angiogenic effects on the angiogenesis process regulating genes and oncogenes. They are presented below (Table 2).

### Epigenetic Compounds

Epigenetic inhibitors that affect the expression of angiogenic factors secreted into the tumor microenvironment have gained interest in developing anti-angiogenic agents. VEGF-induced angiogenesis was analyzed in correlation with HDAC inhibitors known to relieve gene silencing (Table 5).

**TABLE 5:** The most important epigenetic compounds that can affect angiogenesis.

Compounds	Function	References
Trichostatin A	Inhibits histone deacetylases	Deroanne et al., 2002
Suberoylanilide hydroxamic acid	Inhibits histone deacetylases	Marks, 2007
Valproic acid	HDAC inhibitor	Michaelis et al., 2004
Sodium butyrate	HDAC inhibitor	Pellizzaro et al., 2002
5-azacytidine and 5-aza-2′deoxycytidine	DNMT inhibitors	Hellebrekers et al., 2006
Zebularine	DNA methylation inhibitor	Patnaik and Anupriya, 2019
GSK343	Inhibits histone H3K27 methylation	Yu et al., 2017
Panobinostat	Induces acetylation of histone H3	Zhi-Gang et al., 2017
Curcumin	Inhibitor of DNMTs	Hassan et al., 2019
Sulforaphane	Suppress HDACs	Shankar et al., 2008

#### Trichostatin A

Trichostatin A (TSA) has anti-cancer and antifungal effects that selectively inhibits histone deacetylases (HDACs). TSA considerably inhibits the transcription of endothelial receptors such as VEGFR1, VEGFR2 and neuropilin-1, along with the upregulation of SEMA3 (semaphorin 3) in ECs (Deroanne et al., 2002). SEMA3 inhibits angiogenesis and metastatic dissemination of tumor cells by interfering with neuropilin-1-mediated VEGF signaling (Neufeld et al., 2012). It was observed that in the human tongue, squamous cell carcinoma cells have a robust anti-angiogenic activity by reducing HIF-1α protein accumulation and the levels of VEGF mRNA and protein expression (Kang et al., 2012). TSA treatment promotes the stabilization of HIF-1α under normal oxygen conditions, leading to VEGF overexpression. HIF-1α is stabilized and translocated into the nucleus to activate the VEGF promoter by TSA-mediated acetylation at lysine (K) 674. The effectiveness of TSA is reduced by overexpression of HIF-1α or hypoxic conditions, which leads to drug resistance in tumor cells (Lee J. W. et al., 2017). Moreover, it seems that under normoxic conditions, TSA treatment has a concomitant effect on drug resistance and anticancer effects by HIF-1α acetylation (Geng et al., 2011). Trichostatin A is mainly used in clinical trials and also in a phase I clinical trial investigating the safety and tolerability of trichostatin A in adults with relapsed or refractory hematologic malignancies.

#### Suberoylanilide Hydroxamic Acid

Suberoylanilide hydroxamic acid (SAHA) inhibits the histone deacetylases activity of class I and II (HDACs). SAHA selectively inhibits of the pathological development of a variety of transformed cells with relatively little toxicity (Marks, 2007). Several studies have shown that SAHA has an important role in regulating the expression of the VEGFs pathway. It was noted that in lung cancer cell lines, inhibition of HDACs by SAHA and TSA leads to vascular receptors expression reductions such as VEGFR1 and VEGFR2 and their co-receptors neuropilin1 (NP1) and neuropilin2 (NP2). Also, in breast cancer, the HDAC inhibitor SAHA reduces VEGF-C expression in a dose-dependent manner (Cheng and Hung, 2003). Several studies have shown that SAHA sustains autophagy, tumoral cell viability reduction and demonstrates a strong anti-proliferative activity in tumoral cells (Butler et al., 2002; Lee et al., 2012). SAHA exerts selectivity toward HDAC6 and HDAC3. In breast cancer cells, HDAC6 has played a vital role in survivin deacetylation and nuclear export, which is an essential anti-apoptotic agent and thus increases the sensitivity of cells to chemotherapeutic drugs (Lee et al., 2016).

#### Valproic Acid

Valproic acid (VPA) is a HDAC inhibitor, is evaluated for its anti-cancer properties due to its anti-angiogenic potential, inhibits *in vitro* and *in vivo* angiogenesis, involving a reduction in eNOS expression preceded by HDAC inhibition (Michaelis et al., 2004). VPA-treatment significantly inhibits endothelial tube formation and stabilization, nitric oxide production, proliferation, and migration in ECs by promoting EMT states in cancer cells regulating the mesenchymal markers Vimentin-cadherin (Murugavel et al., 2018). Besides the antitumor effect, VPA affects the immune cells by suppressing the inflammatory response mediated by cytokines and oxidative stress molecules (ROS, NO) (Soria-Castro et al., 2019). VPA has an inhibitory effect on pathological retinal angiogenesis in mice through HDAC inhibition, which reduces VEGF expression. In the mouse retina, the mammalian target of rapamycin complex 1 (mTORC1) is activated by VEGF, determining ECs proliferation, leading to angiogenesis (Iizuka et al., 2018).

#### Sodium Butyrate

Sodium butyrate (NaB) induces cell cycle arrest, cancer cell differentiation and apoptosis in colon cancer cells by modulating the expression of VEGF and HIF-1α in a dose-dependent manner (Pellizzaro et al., 2002). NaB suppresses hTERT gene expression and, therefore contributes to a low telomerase activity in human prostate cancer cells (Rahman and Grundy, 2011). NaB is an HDAC inhibitor that reactivates epigenetically silenced genes in cancer cells by promoting apoptosis via p53 and Bax activation and cell-cycle arrest by induction of p21 (Dashwood and Ho, 2007). On the other hand, the inhibition of HDAC by NaB had an anti-inflammatory effect by suppressing nuclear factor κB (NFkB) activation and inhibition of interferon γ production (Hamer et al., 2008).

#### 5-Azacytidine and 5-aza-2′Deoxycytidine

5-Azacytidine and 5-aza-2′deoxycytidine are both nucleoside analogs and DNMT inhibitors which show less toxicity than TSA. 5-Aza-2′-deoxycytidine and TSA reactivate angiogenesis by inhibiting antiangiogenic agents by direct methylation to the promoter of TSP1, JUNB, and IGFBP3 genes, which are suppressed in tumor-conditioned ECs. The effect of these inhibitors is only antiproliferative without affecting ECs, apoptosis and migration and also supporting their angiostatic activity (Hellebrekers et al., 2006).

#### Zebularine

Zebularine is a DNA methylation inhibitor that cooperates with DNMT creating a covalent complex. It shows antiangiogenic activity and significantly reduces vessel development in tumors (Hellebrekers et al., 2006). It has a good correlation between low toxicity and high efficacy, with potential contribution as an adjuvant agent for anticancer treatments and low-dose administration for a prolonged period (Patnaik and Anupriya, 2019).

#### GSK343

GSK343 inhibits histone H3K27 methylation and increases expression of E-cadherin, p21 and PTEN. Also, this drug suppresses the proliferation and tumoral invasion by inhibiting the expression of EZH2 (Yu et al., 2017). The treatment of human umbilical cord blood-derived cells with GSK343 decreases levels of H3K27me2/3 in a time and dose-dependent manner without affecting other histones, including H3K27me1, H3K4me3, H3K9me3 or H4K20me3, which further improves the pro-angiogenic functions (Fraineau et al., 2017).

#### Panobinostat or LBH589

Panobinostat or LBH589 inhibits proliferation and angiogenesis of glioblastoma cells by increasing degradation of HIF-1a mediated by the Hsp90/HDAC6 complex under hypoxic conditions (Zhi-Gang et al., 2017). LBH589 induces acetylation of histone H3 and alpha-tubulin protein on ECs, leading to tumor angiogenesis inhibition. LBH589 represses endothelial tube formation and invasion and inhibits AKT expression, ERK1/2 and CXCR4 *in vitro*. Complementary, in mice LBH589 treatment reduced angiogenesis and tumoral development (Qian et al., 2006).

#### Curcumin (Diferuloylmethane)

Curcumin (diferuloylmethane) is well known as an anti-inflammatory agent, which also exhibits antiangiogenic properties, including downregulation of the proangiogenic factors VEGF, MMP-9, down-regulation of growth factor receptors (EGFR, HER2, etc.) and inhibiting endothelial cell migration and invasion. The epigenetic regulatory roles consist in the inhibition of DNMTs, regulation of HATs, HDACs and miRNA. Curcumin is useful as an agent in cancer chemoprevention in the form of dietary phytochemicals, having the potential role of reversing epigenetic changes and efficient regulation of gene expression that causes tumorigenesis (Hassan et al., 2019). Curcumin is a safe compound with indicated no dose-limiting toxicity at 10 g/day. As a disadvantage, curcumin has poor solubility and a low absorption rate in the gastrointestinal tract after ingestion (Aggarwal et al., 2003).

#### Sulforaphane

Sulforaphane (SFN) is a natural compound found in cruciferous vegetables, which express antitumor effects without any toxic effect, inhibiting tumor growth, metastasis, and angiogenesis. SFN suppress HDACs and mediate epigenetic regulation of apoptosis, cell cycle and inflammation in various cancers (Alumkal et al., 2015). Emerging evidence suggests that SFN downregulate MMP-1, MMP-2, MMP-7, and MMP-9 and therefore intervenes in cancer cells invasion and angiogenesis (Shankar et al., 2008).

## Antiangiogenic Approaches

**FIGURE 3:**
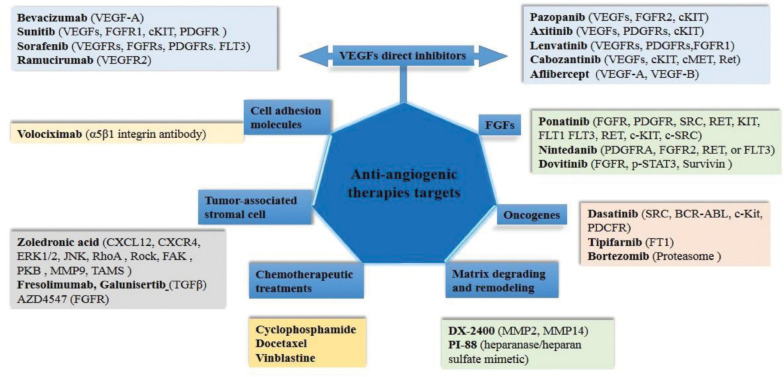
Schematic representation of currently approved anti-angiogenic inhibitors and their targets. Here we delimit the direct VEGF inhibitors and other targets from others, such as FGFs, oncogenes, remodeling and ECM degradation, cell adhesion molecules and conventional chemotherapeutic treatment.

Currently approved anti-angiogenic therapies includes several agents approved for clinical use (Figure 3). Angiogenesis inhibitors were extensively studied in the past decade and resulted in a modest increase in cancer cases’ overall survival when used as monotherapies. Anti-angiogenic therapies focused on VEGF signaling pathways are demonstrating clinical benefits for an increasing number of cancer types. The difference in clinical benefits of treatment practice is manifested when is using multiple chemotherapy drugs. However, only a proportion of patients have a prolonged favorable response to therapy, indicating treatment resistance in some cases (Bergers and Hanahan, 2010).

For a long time, the development of antiangiogenic drugs has focused on VEGF inhibition and its receptors (Figure 3). Bevacizumab is the first FDA approved monoclonal antibody against VEGF-A, which increased the overall survival of patients with metastatic colorectal cancer after 8 months of bevacizumab supplementation in chemotherapy (Cao et al., 2019). Also, sorafenib and sunitinib, tyrosine kinase inhibitors, have shown positive results in liver and kidney cancers (Motzer et al., 2006; Ben Mousa, 2008). In some cases, anti-angiogenic strategies showed no benefit or induced initial responses followed by disease progression, thereby giving limited survival benefit (Bergers and Hanahan, 2010). The failure of this therapeutic approach due to the acquired resistance of the tumors led to an increased understanding of VEGF-independent angiogenesis. Inhibition of VEGF/VEGFR2 has led to the activation of other signaling pathways that support angiogenesis by alternative signaling pathways (Lu et al., 2012). In an attempt to abolish acquired resistance to treatment, trebananib has been proposed as an adjunctive therapy that indirectly inhibits angiogenesis by blocking the interaction between Ang1/Ang2 and the Tie2 receptor. Similarly, cabozantinib can inhibit both the VEGF and c-Met in various solid tumors (Bueno et al., 2017). Moreover, anti-angiogenic therapy may lead to the onset of hypoxia, resulting in the activation of HIF1a in tumor cells with a consequence of tumor cell adaptation to hypoxia and the promotion of tumor angiogenesis (Bergers and Hanahan, 2010).

Clinical findings from bevacizumab, sunitinib, and sorafenib provide valuable information for angiogenesis research, with survival benefits in some aggressive tumors, but in other cases failing to produce lasting clinical responses in most patients including those with metastatic cancer (Mitchell and Bryan, 2010). Treatment failure frequently occurs in patients with metastatic cancer due to various mechanisms that support pathological angiogenesis and acquired resistance, phenomena in which the tumor microenvironment and communication between tumor cells and adjacent non-malignant cells play an essential role (Bueno et al., 2017). Elimination of resistance to therapeutic agents should be evaluated as a dynamic multifactorial process and inhibition of angiogenesis should be optimized by inhibiting alternative signaling pathways and integrating interaction changes between the tumor microenvironment and stromal cells. The incidence of VEGF-resistant tumors targets alternative pathways, which include ANG-1, EGF, FGF, PDGF, and SDF-1 (Bottsford-Miller et al., 2012). Thus, complementary methodologies have been studied, such as combining VEGF inhibitors with agents that target alternative blood vessel formation mechanisms (Figure 3). Volociximab inhibits angiogenesis by blocking the interaction between α5β1 and its ligand fibronectin (Almokadem and Belani, 2012). Bortezomib, the first proteasome inhibitor targeting the ubiquitin-proteasome pathway, has shown a positive clinical benefit both alone and in combination therapy to produce tumor sensitization or to avoid drug resistance for multiple myeloma and mantle cell lymphoma. Bortezomib up-regulate the pro-apoptotic protein NOXA, which interacts with Bcl-2 and results in apoptosis in malignant cells (Chen et al., 2011). Nintedanib, a selective inhibitor of tumor angiogenesis by blocking receptors activities such as VEGFR1–3, PDGFR-α and -β, and FGFR1–3, was proposed to treat non–small cell lung adenocarcinoma and idiopathic lung fibrosis (Hilberg et al., 2018). Also, as a new targeted strategic approach, nanotechnology medical applications have been intensively studied to deliver anti-angiogenic drugs into the tumoral specific sites using nanomaterials as cerium oxide, gold, silver, copper, silica, based on carbon or hyaluronic acid and others (Mukherjee and Patra, 2016).

## Conclusion and Future Directions

Besides their role in normal tissue maintenance, angiogenesis initiation may indicate a shift from tumor latency to malignant active growth and recurrence of the disease. The precise functions of pro- and anti-angiogenic factors and the interactions between them in tumor angiogenesis are not fully understood and the important question is how anti-angiogenic medicine can be improved. However, the mechanisms of induction of vascularization and subsequent development from precancerous lesions to micrometastases achieved by angiogenic strategies for vessel recruitment are not yet fully elucidated in all pathological cases. Specific agents that can block tumor vascularization are required to inhibit angiogenesis and tumor growth.

This review summarizes angiogenic factors involved in each step of vessel development to present an integrated overview of tumor vascularization models (such as cooption, intussusception, sprouting angiogenesis, vasculogenic mimicry, and angioblasts) which, depending on the context, can be helpful for targeted or combined anti-angiogenic therapies. Moreover, we present the epigenetic changes in cancer which in contrast with genetic changes, are potentially reversible, increasing the prospect that epigenetic therapy will be able to mediate tumor fate. In addition to more disease-specific biomarkers, an important issue remains optimization of the dose and frequency of delivery of anti-angiogenic drugs. Current efforts for biomarker discovery in cancer have primarily focused on multi-gene expression patterns, but complementary analysis of DNA methylation signatures may lead to diagnostic and prognostic improvement and better cancer therapy strategies.

The major limitations of drug delivery systems remain the lack of specificity. However, drug-specific therapies that use a lower dose of epi-drugs could improve the effectiveness and tolerability of treatments. Another approach that might improve cancer therapy is the optimization of the dose and duration of release of anti-angiogenic drugs, with potential to alleviate colateral damage that conventional treatments that are toxic to both tumor and normal cells might produce. Future directions for these treatments may include combined drug delivery systems that might target several types of anti-angiogenic factors for synergistic or additive therapeutic effects, and might increase the efficacy and specificity along with reduction of side effects.

## Author Contributions

VA and CD were involved in study conception. IS and CB were involved in study design. VA wrote the manuscript with support from IS, CB, and CD. All authors reviewed and approved the final version of the manuscript.

## Conflict of Interest

The authors declare that the research was conducted in the absence of any commercial or financial relationships that could be construed as a potential conflict of interest.

## Publisher’s Note

All claims expressed in this article are solely those of the authors and do not necessarily represent those of their affiliated organizations, or those of the publisher, the editors and the reviewers. Any product that may be evaluated in this article, or claim that may be made by its manufacturer, is not guaranteed or endorsed by the publisher.
